# An evaluation of methods used to teach quality improvement to undergraduate healthcare students to inform curriculum development within preregistration nurse education: a protocol for systematic review and narrative synthesis

**DOI:** 10.1186/2046-4053-4-8

**Published:** 2015-01-14

**Authors:** Lorraine Armstrong, William Lauder, Ashley Shepherd

**Affiliations:** School of Health Sciences, University of Stirling, Stirling Campus, Stirling, FK9 4LA UK; School of Health Sciences, University of Stirling, Highland Campus, Centre for Health Science, Old Perth Road, Inverness, IV2 3JH UK

**Keywords:** Systematic review protocol, Quality improvement, Curriculum, Education, Preregistration nursing, Teaching methods, Evaluation, Narrative synthesis

## Abstract

**Background:**

Despite criticism, quality improvement (QI) continues to drive political and educational priorities within health care. Until recently, QI educational interventions have varied, targeting mainly postgraduates, middle management and the medical profession. However, there is now consensus within the UK, USA and beyond to integrate QI explicitly into nurse education, and faculties may require redesign of their QI curriculum to achieve this. Whilst growth in QI preregistration nurse education is emerging, little empirical evidence exists to determine such effects. Furthermore, previous healthcare studies evaluating QI educational interventions lend little in the way of support and have instead been subject to criticism. They reveal methodological weakness such as no reporting of theoretical underpinnings, insufficient intervention description, poor evaluation methods, little clinical or patient impact and lack of sustainability. This study aims therefore to identify, evaluate and synthesise teaching methods used within the undergraduate population to aid development of QI curriculum within preregistration nurse education.

**Methods/design:**

A systematic review of the literature will be conducted. Electronic databases, Cumulative Index to Nursing and Allied Health Literature (CINAHL), Psychological Information (PsychINFO), Education Resources Information Centre (ERIC), Medical Literature Analysis and Retrieval System Online (MEDLINE) and Applied Social Sciences Index and Abstracts (ASSIA), will be searched alongside reference list scanning and a grey literature search. Peer-reviewed studies from 2000–2014 will be identified using key terms quality improvement, education, curriculum, training, undergraduate, teaching methods, students and evaluation. Studies describing a QI themed educational intervention aimed at undergraduate healthcare students will be included and data extracted using a modified version of the Reporting of Primary Studies in Education (REPOSE) Guidelines. Studies will be judged for quality and relevance using the Evidence for Policy and Practice Information and Co-ordinating Centre’s (EPPI) Weight of Evidence framework and a narrative synthesis of the findings provided.

**Discussion:**

This study aims to identify, evaluate and synthesise the teaching methods used in quality improvement education for undergraduate healthcare students where currently this is lacking. This will enable nursing faculty to adopt the most effective methods when developing QI education within their curriculum.

**Systematic review registration:**

Prospero CRD42014013847

## Background

Quality improvement (QI) has no single definition but can be conceptualised as an umbrella term which encompasses many different systematic ‘change methods’ to support improvement and better outcomes for patients and services [1]. It remains high on the UK political and educational agenda, continuing to be a key priority within the healthcare system [2, 3].

The first signs of a ‘quality’ boom in health care could be seen at the start of the millennium when the Institute of Medicines’ advancing approaches to innovation and improvement was publicised through *Crossing the Quality Chasm* [4]. Almost a decade later, Scotland adopted a similar approach, making clear their intentions to transform the NHS through The Healthcare Quality Strategy [2]. Despite both influential policy documents highlighting that a) an ‘*all*’ healthcare professionals approach would be necessary and b) a bottom-up approach was required; disappointingly, translation of this message within the nursing profession has shown slow progress.

That is not to say however that a lack of effort or educational resources exists; quite the opposite. For one, many faculties offer postgraduate education at Diploma or Masters Level for practitioners albeit at a cost [3]. The Institute of Healthcare Improvement has alternatively developed a range of free Patient Safety and QI e-learning modules for students and staff to access through their Open School [5]. Building on this further, the World Health Organization (WHO) have well established ‘ready-to-teach’ programmes for faculties to utilise within their curriculum alongside a selection of learning resources; [6] as have the 1000 Lives Plus Campaign in NHS Wales [7]. In Scotland, the Quality Improvement Hub provide a free national platform, home to multiple resources such as case studies, e-learning modules and even a curriculum framework. Here, staff are able to identify what skills, knowledge and behaviours are conducive to QI [8]. It raises an important question therefore as to why, when there are scrupulous resources available, that transition of a QI culture within the nursing profession has been slow to develop.

Empirically, evidence has suggested that lack of staff engagement and time may be key factors [9]. Likewise, no gold standard QI curriculum exists for preregistration nurses. Instead, QI content remains rather implicit, varying greatly between faculties with regard to content and quantity and is potentially subject to dissimilarities in academic staff knowledge [10, 11]. Furthermore, the choice of teaching methods employed by individual faculties remains discretionary and at best selected, understandably, on the resources available. However, faculty my take a different approach to the choice of teaching methods adopted if there was a suitable evidence base. The research literature [3] and an additional scoping review only magnify the lack of studies evaluating teaching methods for QI nurse education. If we are to create the capacity and capability of a future workforce both skilled and knowledgeable in improving the care of our patients and services, then surely the impact of different teaching methods should be fully understood?

The scoping review carried out to explore this further has necessitated greater need for a comprehensive systematic review of the literature. Although there is an increasing body of literature in QI nurse education, lack of empirical evidence exists to support or inform effective curriculum development [12–14]. Instead, a selection of previous systematic reviews of QI educational interventions exists and targets mainly large collaboratives (multi-organisational and multi-disciplinary teams who facilitate large-scale QI initiatives), middle management, the acute sector or the postgraduate medical profession [3, 15–22]. The focus of these studies includes attitudes of medical students to inform curriculum development [15], non-technical skills training in acute areas [22], factors that promote or hinder curricular implementation [23] and effects of teaching QI to clinicians and senior doctors [19, 20]. Whilst these studies do confirm some improvement in learner knowledge [20, 23], no changes have been identified in learner behaviour, organisational or patient outcomes [22, 23]. Furthermore, these studies are criticised for poor study design, utility of invalidated assessment tools, lack of intervention description, little use of longitudinal evaluation methods and no theoretical underpinnings [20, 22].

The latter is surprising in that the Medical Research Council (MRC) advocate that a strong theoretical underpinning is a key to any intervention success [24]. For example, employing the philosophical assumption of constructivism would suggest that students who engage in *active* learning processes, as opposed to ones that are *passive*, will translate knowledge more effectively into practice [25]. One could proclaim therefore that teaching methods composing a practical element may be more effective in teaching QI. That being said, this philosophy lays only the foundations to a theoretical framework and our review wishes to build on this further by adopting the use of two closely related impact theories. Impact theories set the expectations of how an intervention is likely to enable change, detailing potential causes and effects as well as highlighting the facilitating and hindering factors that may be involved [26].

Experiential learning is the first example. This educational theory most associated with David Kolb targets individuals to improve skills, knowledge and attitudes by the process of learning from direct experience and reflection [27]. Kolb’s Experiential Learning Model presents a four-stage cyclical process that allows learners to 1) be actively involved in the experience, 2) reflect on the experience, 3) utilise analytical thinking and 4) make decisions and solve problems using new ideas [27]. This model bears close resemblance to that of Langley et al. and their Model for Improvement [28]. The Model for Improvement, although a two-part model consists of rapid cycle changes called Plan-Do-Study-Act. The ‘plan’ phase requires individuals to know the who? what? when? where? and what data to collect—which indicates that individuals would need to be actively involved to do this. The ‘do’ and ‘study’ phase requires both analysis of data and reflection on what was learned from each cycle, leaving the ‘act’ phase to determine (from that data) what modifications can be made [28]. Due to the similarities of these two models, it would seem indicative that teaching QI to undergraduate students using an experiential learning teaching method could be extremely effective; but this is not necessarily correct in all cases.

The second example of Social Learning theory highlights this well. Bandura’s Social Learning Theory suggests that learning occurs in social contexts through continuous interactions with others by the process of observation. This means that learning may occur as a result of observing good behaviour demonstrated by a group or individual but equally as a result of the consequences of poor behaviour; this process is called ‘*modelling*’ [26]. Understanding and combining Experiential Learning and Social Learning Theory may assist in our understanding of how QI educational interventions may work (or not) and allow us to formulate an appropriate hypothesis.

For example, considering both impact theories, a hypothesis that experiential learning would impact most positively on students’ skills knowledge and attitude could be made. However, the influence of observed behaviours in the social learning context may dictate whether a positive or negative impact on student behaviour occurs. James et al. [29] and The Health Foundation [30] support this view by asserting that QI should not be limited to the theoretical learning of technical skills involved (PDSA cycles and run charts) but should include the soft skills (social psychology of change), the learning skills (critical reflection, action learning) and indeed the interactions between them.

### Study aims and objectives

The aim of this study therefore is to systematically review and evaluate the educational methods used to teach QI to undergraduate healthcare students.

Study objectives are to:

1. Identify and describe the variety of teaching methods used in QI education for undergraduate healthcare students.
2. Determine what teaching methods impact most positively on two primary outcome measures: skills, knowledge and attitude and behaviour.
3. Determine what teaching methods impact most positively on two secondary outcome measures: student reaction and patient outcomes.
4. Identify factors that promote or hinder the effectiveness of teaching methods in skills, knowledge and attitude and behaviour.

## Methods/design

### Study design

This study will be guided and conducted using the Evidence for Policy and Practice Information and Co-ordinating Centre’s (EPPI) approach to systematic reviewing and Guidance on the Conduct of Narrative Synthesis in Systematic Reviews [31, 32]. This approach will enable a rigorous process to be followed ensuring that both methodological decisions taken remain appropriate to the review type and anticipated data set.

### Study methods

#### Search strategy

To identify studies relevant to the review question, a range of databases will be searched from Education, the Social Sciences and Health care.

##### Electronic databases

Electronic databases, Cumulative Index to Nursing and Allied Health Literature (CINAHL), Psychological Information (PsychINFO), Medical Literature Analysis and Retrieval System Online (MEDLINE), Science Direct, Education Resources Information Centre (ERIC), Applied Social Sciences Index and Abstracts (ASSIA), will be systematically searched. Study limitations will ensure that only peer reviewed and English language studies are retrieved. As the quality healthcare boom became most apparent in the millennium, databases will be searched from 2000–2014. As per EPPI Guidelines, a primary search will be developed in MEDLINE using Medical Subject Headings (MeSH) terms [31]. Subsequent searches will be translated through identification and selection of controlled terms specific to each database and Boolean terms, truncation, wildcards and proximity searching will be applied where necessary to maximise relevant hits. Search terms will comprehensively include quality improvement, improvement science, science of improvement, continuous quality improvement, total quality management, quality standards, improvement models, education, training, teaching, learning, course, curriculum, curriculum development, student(s), trainee(s), undergraduate, evaluation and programme evaluation. A primary sample search strategy from MEDLINE is presented (see Table 1).

**Table 1:** **Developed search strategy for MEDLINE using OVID**

	Search terms
1.	exp quality Improvement*/
2.	(science of improvement or improvement science or continuous quality improvement or total quality management or quality standards or improvement models).tw.
3.	1 or 2
4.	exp education*/
5.	course$.tw.
6.	train$.tw.
7.	curricul$.tw.
8.	teach$.tw.
9.	learn$.tw.
10.	4or 5 or 6 or 7 or 8 or 9
11.	student.ab.
12.	trainee.ab.
13.	learner.ab.
14.	(undergraduate).ab.
15.	11 or 12 or 13 or 14
16.	exp programme evaluation/
17.	evaluat$.ab.
18.	16 or 17
19.	3 and 10 and 15 and 18

The ‘exp’ before an index term indicates that the term was exploded. The slash (/) after an index term indicates that all subheadings were selected. ‘tw’ or ‘ab’ indicates a search for a term in the title (ti) or abstract (ab). The dollar ($) at the end of a term indicates that this term has been truncated. The asterisk (*) following an index term indicates that that term was focused—i.e. limited to records where the term was a major MeSH term.

##### Reference list searching

Eligible studies will have a full scan of their reference list carried out to identify any potentially relevant studies not detected initially by the search strategy. Where difficulties in retrieving studies are encountered, authors will be contacted to request a copy of the article.

##### Grey literature

Websites of leading organisations and institutions, within the field of QI will be searched for published or unpublished materials relevant to the review question. Where a search engine is available, keywords will be input individually or combined if this option is available. Otherwise, websites will be searched manually.

### Inclusion/exclusion criteria

Inclusion:

As presented in Table 2, we will include all peer-reviewed (primary) studies of all designs that have an available abstract written in the English language from 2000 (or inception, if later) until 2014. Studies will be included if they describe a QI educational intervention targeting undergraduate healthcare students only. We will accept and include any ‘quality improvement educational intervention’ as one that describes the teaching, learning or utility of one of the five main healthcare models: total quality management, continuous quality improvement, business process reengineering, Institute of Healthcare Improvement rapid cycle change (Model for Improvement) and lean thinking or six sigma [33].

**Table 2:** **Screening criteria for study inclusion/exclusion to review**

Criteria			
	Yes	No	Unsure
Peer-reviewed article			
English language			
Abstract available			
Within geographical subset			
Primary/original data			
QI education			
Undergraduate healthcare student			
Evaluative outcome			

Exclusion:

We will exclude studies that describe both pre- and post-university educational interventions, describe only patient safety content, do not document original data or do not fall within the predetermined dates. We will exclude studies where the educational intervention does not include undergraduate students nor has an evaluative outcome. As previous studies have retrieved most results from the UK, USA, The Netherlands, Scandinavia, Canada and Australasia, any studies found out with this geographical subset will be excluded [3].

### Data collection and management

#### Search

One researcher will conduct and save each search within corresponding databases. To ensure that each search is accessible, reproducible and transparent, a manual record keeping log will be produced. To allow reviewer access, studies eligible for screening will be uploaded electronically or manually to a shared named folder within reference management software (Refworks). Duplicates at this stage will be removed.

#### Screening and selection of studies

Two reviewers will work independently to screen titles and abstracts from retrieved studies using predetermined inclusion and exclusion criteria (see Table 2). Items that fall within the category of ‘unsure’ will be discussed and the decision to include or exclude will be made jointly. Articles for inclusion will be transferred to a separate shared named folder to allow reviewer access for data extraction. A portion of excluded studies may hold value in that data can be utilised either for discussion within this study or future studies. Therefore, articles excluded (solely) by one of the exclusion criteria (see Table 3) will be stored in Refworks, coded appropriately and be given a textual description, as recommend by Gough et al. [31] To ensure consistency in the application of inclusion and exclusion criteria both reviewers will screen a 10% random sample of the same papers and inter-rater reliability will be confirmed. Any discrepancies will be resolved by discussion and a third reviewer where necessary. The results yielded from each phase of the search strategy will be illustrated using the PRISMA flow diagram [34].

**Table 3:** **Exclusion criteria and codes**

Exclusion	Code
No original/primary data	A
Targets different population	B
Outwith geographical subset	C

#### Data extraction

Two reviewers will be guided but not restricted by a modified version of Reporting of Primary Studies in Education (REPOSE) Guidelines when extracting data from studies [35]. Where information is incomplete, attempt to contact authors for clarification will be sought (see Table 4). Although highly criticised, our initial scoping review identified several intervening variables that may have contributed to hindering or facilitating a successful outcome and included: support structures and curriculum demand [15], feedback mechanisms and group size [13] and practice observation, shortages in practice staff and student/tutor relationships [11]. We will therefore attempt to extract facilitating or hindering factors where either these are explicitly reported by study authors or where the reviewers identify implicitly a relation to our two impact theories and hypothesis, e.g. observed positive or negative behaviour. Outcomes of interest will be extracted according to a model recommended by Parry et al. called The Kirkpatrick Model which uses a four-tier framework to evaluate training programmes [36]. This includes level 1) Reaction; how did the learners react to the learning experience? Was it enjoyable? 2) Learning; what knowledge, skills and attitudes have they acquired as a result? 3) Behaviour; changes in professional practice? and 4) Results; patient outcomes [37]. These will be reported as simple statistics as reported by study authors. Reviewers will compare a 10% random sample to ensure consistency, and discrepancies will be resolved by discussion or third reviewer where necessary.

**Table 4:** **Data extraction sheet modified from REPOSE Guidelines**

Study characteristics	
General information	Article title
	Author name(s)
	Publication date
	Country of origin
	Discipline
Introduction	Study aims and rationale
	Study research question(s)
	Theoretical underpinning
Methods	Research design
	Sample strategy
	Outcome measure(s):
	Data collection/analysis
	Evaluation model/method
Intervention	Type of learner(s)
	Intervention description
	Content
	Teaching method
	QI model used
	Learning environment
	Group size
Outcome measures	(1) Knowledge, skills and attitude/behaviour
	(2) Student reaction/patient outcomes
Facilitating factors	e.g. support structures
Hindering factors	e.g. lack of resources
Outcome/results	Follow up
	Author’s conclusion

#### Quality and relevance

This review is predicted to retrieve heterogeneity in both study design and outcomes therefore using a singular hierarchical appraisal tool would not be fit for purpose nor would it be beneficial in answering the review questions [31]. Instead, assessing the evidence will follow the EPPI Weight of Evidence framework which considers the use of judgement in relevance and quality. This framework allocates each study with a weight of high, medium or low in relation to three key areas: a) trustworthiness of results of study, b) appropriateness of study design to review question and c) appropriateness of focus to answering review question. The results of a), b) and c) are combined and given an overall weight. For individual studies to be given a fair critique, it is recommended that incorporating the TAPUPAS framework created by Pawson et al. may be beneficial (see Table 5) [38]. To ensure consistency in overall Weight of Evidence allocated, reviewers will meet to compare a 10% random sample of low-, medium- and high-graded papers and discrepancies resolved through discussion or a third reviewer if necessary. Low-graded papers will be excluded from the final synthesis.

**Table 5:** **Weight of Evidence and TAPUPAS for quality and relevance of studies**

Weight of Evidence	TAPUPAS dimensions
A = Trustworthiness of results of study (methodological quality)	(T) Transparency (A) Accuracy (A) Accessibility (S) Specificity
B = Appropriateness of study design to review question (methodological relevance)	(P) Purposivity
C = Appropriateness of focus to answer review question (topic relevance)	(U) Utility (P) Propriety
D = Overall Weight of Evidence (based on A, B, C)	Low, medium or high

### Data synthesis

As a general framework, Guidance on the Conduct of Narrative Synthesis in Systematic Reviews has been applied [32]. This non-linear approach comprises of four key stages: (1) developing a theory of how the intervention may work (e.g. combining our two impact theories to formulate a hypothesis), (2) developing a preliminary synthesis of the findings of included studies, (3) exploring relationships in the data and (4) assessing the robustness of the synthesis (e.g. applying the EPPI Weight of Evidence framework).

To develop a preliminary synthesis (2), we will display all characteristics and results of all eligible studies through tabulation. As a means of simplifying the process and aiding subsequent analysis, we will then group individual studies by population (e.g. nursing or medicine) to allow easier identification of patterns emerging within and between studies and/or disciplines. Tabulated data here will include study design, intervention description, teaching methods, outcome measures and effect. We will organise facilitating and hindering factors through thematic analysis, including factors as a theme where it appears more than once across studies. We will apply vote counting by assigning each factor one vote every time it is presented across studies to calculate the frequency of results. A summary of each theme will be reported through tabulation and a brief textual description.

Due to the expected variability in outcomes and heterogeneity in design, a meta-analysis would not be appropriate. Therefore, we will conduct a subgroup analysis using levels 2 and 3 of Kirkpatrick Model [37] as a framework and will explore relationships in the data (3) through concept mapping. This method allows a visual representation of possible cause and effect relationships between the data by using flow charts and diagrams. Findings will be synthesised through a textual narrative directed by the review objectives.

## Discussion

QI continues to dominate all aspects of healthcare delivery. Although it is emerging as a taught component in preregistration nurse education, little empirical research exists to inform the most effective way to teach QI to undergraduate healthcare students. Identifying, evaluating and synthesising teaching methods as well as awareness of the key facilitating and hindering factors associated have potential to aid nursing faculties in the development of QI education, where currently this is lacking.

## Authors’ information

LA is a recent Clinical Academic Fellow conducting a PhD within the field of Quality Improvement and was involved as an undergraduate nursing student in piloting the Institute of Healthcare Improvement Quality Improvement Practicum at University of Stirling.

## Abbreviations

ASSIA: Applied Social Sciences Index and Abstracts
CINAHL: Cumulative Index to Nursing and Allied Health Literature
EPPI: Evidence for Policy and Practice Information and Co-ordinating Centre
ERIC: Education Resources Information Centre
IHI: Institute of Healthcare Improvement
MEDLINE: Medical Literature Analysis and Retrieval System Online
PsychINFO: Psychological Information
QI: Quality improvement.
